# Modelling the impact of different irrigation regimes and mulching on strawberry crop growth and water use in the arsenic-contaminated Bengal basin

**DOI:** 10.1038/s41598-024-56664-4

**Published:** 2024-04-26

**Authors:** Benukar Biswas, Tridiv Ghosh, Debashis Chakraborty, Saon Banerjee, Baidya Nath Mandal, Sarathi Saha

**Affiliations:** 1 Bidhan Chandra Krishi Viswa Vidyalaya, Faculty of Agriculture, Mohanpur, Nadia, West Bengal 741 252 India; 2 Division of Agricultural Physics, Indian Agricultural Research Institute, New Delhi, 110 012 India; 3 International Maize and Wheat Improvement Center (CIMMYT), New Delhi, 110 012 India; 4 ICAR-Indian Agricultural Statistics Research Institute, New Delhi, 110 012 India

**Keywords:** Climate sciences, Ecology, Environmental sciences

## Abstract

Replacement of water-intensive winter rice with strawberry (*Fragaria* × *ananassa* Duch.) may restrict groundwater extraction and improve water productivity and sustainability of agricultural production in the arsenic-contaminated Bengal basin. The potential of strawberry cultivation in terms of yield obtained and water use efficiency need to be evaluated under predominant soil types with mulch applications. Water-driven model AquaCrop was used to predict the canopy cover, soil water storage and above-ground biomass of strawberry in an arsenic-contaminated area in the Bengal basin. After successful calibration and validation over three seasons, AquaCrop was used over a range of management scenarios (nine drip-irrigation × three soil types × four mulch materials) to identify the best irrigation options for a drip-irrigated strawberry crop. The most appropriate irrigation of 176 mm for clay loam soil in lowland and 189 mm for sandy clay loam in medium land rice areas and the use of organic mulch from locally available jute agrotextile improved 1.4 times higher yield and 1.7 times higher water productivity than that of without mulch. Strawberry can be introduced as an alternative crop replacing rice in non-traditional upland and medium land areas of the arsenic-contaminated Bengal basin with 88% lower groundwater extraction load and better economic return to farmers.

## Introduction

The Bengal basin with about 200,000 km^2^ is spread over most of Bangladesh and some parts of India and supports > 2% of the global population^1^*.* It is the world’s largest fluviodeltaic basin with deposition of 22 km thickness of syn-orogenic sediment through the Ganges–Brahmaputra-Meghna river system. The lowland areas (about 30mabove mean sea level-amsl) in the basin are predominantly clayey in texture, while the medium (31–60 m amsl) and upland (> 60 m amsl) soils are clay loam and sandy clay loam, respectively^2^.

The primary constraint faced by the agricultural sector in this region is the water scarcity^3,4^. Growing winter rice in the dry season in non-traditional medium- and upland areas led to an over-exploitation of groundwater^5^, and eventually, a sharp decline in groundwater was recorded (0.92 m decline over 2013–2019;^6^) in the region. This was accompanied by a large increase in arsenic bioavailability over the years^1^. A large human population has been exposed to high arsenic content in groundwater, making it the most severe arsenic-contaminated area in the world with multiple health hazards and socio-economic consequences. Various interventions including water management practices like alternative irrigation with aerobic water management^7^, maintaining aerobic conditions^8^ and sprinkler irrigation^9^; application of amendments like organic matter^10^, biochar^11^, balanced fertilizers^12^, phosphate^13^, combination of nitrogen and iron^14^, nitrate fertilizer^15^, silicon^16^ and sulfur^17^ have been investigated but these have limited efficiency in field condition due to complex behavioural pattern of the toxic metal^18^. Adak (2002)^19^ recommended the cultivation of sesame, green gram, and mustard in regions with limited arsenic-contaminated water, serving as an alternative to winter rice. Diversification of crops suited to the area's environmental conditions is essential, emphasizing nutritional value and high market prices. Strawberries are highlighted for their rich content of vitamin C, potassium, and fibre, along with antioxidants like ellagic acid. This compound prevents arsenic-induced myofibrillar loss and coagulative necrosis, combating toxicity and carcinogenicity caused by oxidative stress, particularly in malnourished populations of the Bengal basin. Growing day-neutral strawberry varieties in sub-tropical plains during winter is suggested for economic value as fresh fruit or in processed products like jams and ice cream^20–26^. One hectare of land with strawberry cultivation may provide a return of 2358 USD, compared to 662 USD return from winter rice^5^. Partial replacement of rice in non-traditional areas of Bengal basin with drip-irrigated strawberry decreased arsenic bioavailability and it could be an alternative mitigation option^5^. Strawberry is a water-sensitive fruit crop with a high risk of phytophthora root rot and other diseases under excess irrigation and yield reduction under water deficit conditions^5^. An optimum soil water regime in strawberry needs to be devised through precise irrigation scheduling and the use of cover mulch to conserve soil water. Mulching increases strawberry yield and nutrient use efficiency while preserving soil water, regulating soil temperature, and reducing weed and insect pest infestation^27^. However, poorly reversible plastic mulches have serious environmental and public health issues^28^. Here, three organic mulch materials biodegradable polymers^29^, agrotextile mulch made by locally available jute fiber^30^ and rice straw were evaluated.

Crop growth simulation models, including WOFOST, CERES-Barley, HERMES, DAISY, and AquaCrop, have been effective tools for evaluating the impact of irrigation management on crop growth and performance^31^. These models play a crucial role in estimating crop water requirements, yield, and water productivity across diverse scenarios. AquaCrop, in particular, has been applied by researchers^32^ to assess the effects of irrigation management on maize yield, leading to the identification of optimal strategies for maximizing water productivity. The combination of models using SWAT and MODSIM, enhances the accuracy of water productivity assessments for crops like wheat and maize in specific catchment areas^33^. However, considering factors such as complexity, accuracy, and parameter requirements, AquaCrop, with its water-driven growth module, emerges as a suitable choice for simulating crop growth. This model is particularly well-suited for addressing conditions where water is a critical limiting factor in crop production^34^. This water-driven model for simulating crop water productivity developed by the Food and Agriculture Organization, could be applied for optimum irrigation scheduling of a crop by normalizing the water productivity parameter for evaporative demand and atmospheric CO_2_ concentration^35^. AquaCrop has been previously used over a wide range of agro-ecological zones for various herbaceous crops such as quinoa, bambara groundnut, maize, soybean, amaranthus, pea, rice, cabbage, barley, teff, sugarcane, and cotton^36^. To date, no study on simulation of the effects of deficit irrigation with drip and mulch application on strawberry with the AquaCrop model has been reported in the literature. The purpose of this study was to test the performance of the AquaCrop model in simulating the growth and production of strawberry through proper calibration and validation. The performance analysis of drip-irrigated strawberry under different irrigation regimes and mulching option is another objective of the present research paper.

## Results

### Model calibration

The initial (CC_o_) and maximum (CC_m_) canopy cover by strawberry with a population of 5.3 m^−2^ were 0.80 (80%) and 95% respectively at 1.0ETc under the straw mulching. Calibrated coefficients of canopy growth (CGC) and canopy decline (CDC) were measured as 10.4% d^−1^ and 8.0% d^−1^, respectively. Average soil moisture at the active root zone (0.40 m) was around two times higher at drip-irrigated mulched plots over unmulched surface irrigated one (Supplementary Fig. S3). The calibrated aboveground biomass WP value was adjusted to 17 g m^−2^, which was within the model-recommended range of 15–20 g m^−2^ for C3 plants. The value of the crop sink-strength coefficient (f_sink_) was set at 40%^37,38^, while the value of the reduction coefficient for the products synthesized (f_yield_) was taken as 50%. The reference harvest index (HI_o_) for the Sweet Charlie variety in the study was estimated at 35%^39^.

The threshold values of soil water depletion were adjusted at 0.20 to 0.55 for canopy expansion, 0.50 for stomatal conductance, and 0.65 for canopy senescence as per model guidelines^35,40^. These adjustments were made keeping in mind the shallow root distribution of strawberry under mulch and drip irrigation and making the growth sensitive to the soil water (Table 1).

**Table 1:** Date of planting and irrigation scheduling of strawberry in different years.

Year	Planting date	Irrigation treatment^1^	Number of irrigations	Total irrigation amount (mm)^2^
2015–16	02-October	0.6ETc	15	115
		0.8ETc	15	145
		1.0ETc	15	175
2016–17	01-October	0.6ETc	7	67
		0.8ETc	7	81
		1.0ETc	7	95
2017–18	18-October	0.6ETc	14	109
		0.8ETc	14	137
		1.0ETc	14	165

^1^Drip irrigation to meet 100% (1.0ETc), 80% (0.8ETc) and 60% (0.6ETc) crop evapotranspiration (ETc), respectively, under the standard condition^67^ [ETc = E-pan × Pan coefficient (Kp, 0.85) × crop coefficient (Kc)]; *Kc values were taken from FAO manual No. 56^67^.

^2^Includes 25 mm water for field preparation and establishment of seedlings.

Calibrated parameters (Table 2) indicated good agreements between observed and model-simulated data. The r^2^ values were 0.94–0.98 for canopy cover, 0.92–0.99 for soil–water-storage in the root zone, 0.81–0.99 for aboveground biomass, and 0.90 for yield. The corresponding RMSE was measured as 2.3–6.7%, 8.3–14.9 mm, 7.9–16.2, and 1.1 t ha^−1^ and d values at 0.96–0.99, 0.88–0.97, 0.90–0.99, and 0.96, respectively.

**Table 2:** Calibration statistics for the canopy cover, soil water storage, aboveground biomass and yield of strawberry under different irrigation regimes and mulching during 2015–16.

Indicator	Treatment	n	r^2^	RMSE	d
Canopy cover (%)	0.6ETcNM	3	0.98	2.6	0.99
	0.6ETcSM	3	0.96	4.9	0.98
	0.6ETcBM	3	0.98	4.2	0.99
	0.6ETcJM	3	0.98	4.2	0.99
	0.8ETcNM	3	0.98	6.4	0.96
	0.8ETcSM	3	0.96	2.3	1.00
	0.8ETcBM	3	0.96	4.3	0.99
	0.8ETcJM	3	0.94	6.4	0.95
	1.0ETcNM	3	0.98	3.7	0.99
	1.0ETcSM	3	0.98	4.1	0.99
	1.0ETcBM	3	0.98	5.5	0.99
	1.0ETcJM	3	0.94	6.7	0.97
	Pooled over the treatments	36	0.97	2.3	0.90
SWS (mm)	0.6ETcNM	3	0.92	11.6	0.92
	0.6ETcSM	3	0.98	13.7	0.89
	0.6ETcBM	3	0.99	14.9	0.88
	0.6ETcJM	3	0.94	14.6	0.88
	0.8ETcNM	3	0.98	8.5	0.94
	0.8ETcSM	3	0.99	8.3	0.97
	0.8ETcBM	3	0.99	9.6	0.96
	0.8ETcJM	3	0.98	14.0	0.92
	1.0ETcNM	3	0.99	10.2	0.92
	1.0ETcSM	3	0.98	13.6	0.93
	1.0ETcBM	3	0.99	12.2	0.95
	1.0ETcJM	3	0.98	14.2	0.93
	Pooled over the treatments	36	0.96	13.0	0.54
Aboveground biomass (t ha^−1^)	0.6ETcNM	3	0.99	10.0	1.00
	0.6ETcSM	3	0.81	11.3	0.90
	0.6ETcBM	3	0.98	12.7	0.99
	0.6ETcJM	3	0.99	12.9	1.00
	0.8ETcNM	3	0.94	13.2	0.98
	0.8ETcSM	3	0.96	16.2	0.98
	0.8ETcBM	3	0.99	13.7	0.99
	0.8ETcJM	3	0.92	16.2	0.97
	1.0ETcNM	3	0.92	7.9	0.97
	1.0ETcSM	3	0.90	11.2	0.97
	1.0ETcBM	3	0.99	8.9	0.99
	1.0ETcJM	3	0.94	11.7	0.98
Yield (t ha^−1^)	Pooled over the treatments	36	0.90	1.1	0.96

n, r^2^, RMSE, and d indicate the number of observations, coefficient of determination, root mean square error, and the index of agreement respectively. NM, SM, BM and JM are no-mulch, straw mulch, biodegradable plastic and jute agrotextile mulch, respectively. Drip irrigation treatments to meet 100, 80 and 60% crop evapotranspiration (ETc) are 1.0ETc, 0.8ETc and 0.6ETc, respectively.

### Model validation

#### Canopy cover

The model satisfactorily simulated the seasonal trend in canopy cover in all treatments for two consecutive seasons with r^2^ of 0.94, RMSE of 4.8%, and d of 0.94 (Supplementary Fig. S5 and Table 3). The model captured the variability of canopy cover in all the irrigation-mulch combinations except during peak vegetative stage to maturity (45–140DAP) in 0.8ETcNM, 0, 8ETcBM and 1.0ETcJM in 2016–17 (Supplementary Fig. S4a) and in 0.8ETcNM, 1.0ETcSM and 1.0ETcBM during early-to-peak flowering stage (45–75DAP) in 2017–18 (Supplementary Fig. S4b). In all these cases, model values were 6.5% lower than the field-measured values but the canopy senescence was appropriately estimated in all the treatments during both seasons.

**Table 3:** Validation statistics for the canopy cover, aboveground biomass and soil water storage (over the 400 mm profile) in strawberry under different irrigation regimes and mulching during 2016–17 and 2017–18 seasons.

Year	Treatment^1^	Canopy cover				Aboveground biomass				Soil water storage			
		n	r^2^	RMSE (%)	d	n	r^2^	RMSE (t ha^−1^)	d	n	r^2^	RMSE (mm)	d
2016–17	T1	3	1.00	4.1	0.99	3	0.95	0.4	0.94	3	0.88	9.2	0.94
	T2	3	0.99	6.0	0.98	3	1.00	0.2	0.99	3	0.92	13.6	0.89
	T3	3	1.00	6.6	0.97	3	0.99	0.2	0.99	3	0.90	21.0	0.77
	T4	3	1.00	5.8	0.99	3	1.00	0.4	0.98	3	0.94	10.9	0.92
	T5	3	0.99	8.2	0.94	3	0.95	0.4	0.97	3	0.93	15.1	0.84
	T6	3	0.98	4.2	0.99	3	0.96	0.6	0.95	3	0.97	16.5	0.89
	T7	3	1.00	7.1	0.97	3	0.97	0.5	0.98	3	0.96	14.8	0.91
	T8	3	0.99	7.6	0.97	3	0.97	0.5	0.98	3	0.95	16.5	0.87
	T9	3	0.99	3.6	0.99	3	0.97	0.5	0.97	3	0.99	13.2	0.89
	T10	3	0.97	6.1	0.98	3	0.96	0.6	0.97	3	0.97	15.6	0.90
	T11	3	0.96	8.9	0.97	3	0.96	0.8	0.96	3	0.98	16.3	0.92
	T12	3	0.99	7.4	0.98	3	0.95	0.8	0.97	3	0.98	12.0	0.94
2017–18	T1	3	0.96	6.8	0.96	3	0.96	0.3	0.97	3	0.66	12.9	0.88
	T2	3	1.00	4.1	0.99	3	0.96	0.6	0.96	3	0.66	16.1	0.85
	T3	3	0.98	5.3	0.98	3	0.98	0.3	0.99	3	0.74	19.4	0.76
	T4	3	0.98	3.6	0.99	3	0.98	0.4	0.97	3	0.90	12.3	0.91
	T5	3	0.96	3.8	0.98	3	1.00	0.2	0.99	3	0.94	7.4	0.95
	T6	3	0.98	3.2	0.99	3	0.94	0.5	0.98	3	0.96	15.4	0.90
	T7	3	0.98	3.2	0.99	3	0.94	0.5	0.98	3	0.96	18.2	0.88
	T8	3	0.96	5.1	0.98	3	0.96	0.5	0.99	3	1.00	17.3	0.83
	T9	3	0.96	4.5	0.98	3	0.92	0.6	0.96	3	0.81	14.5	0.84
	T10	3	0.86	9.7	0.96	3	0.92	0.7	0.94	3	0.94	12.0	0.94
	T11	3	0.94	11.3	0.96	3	0.92	0.8	0.96	3	0.86	13.2	0.92
	T12	3	0.98	4.2	0.99	3	0.86	0.9	0.96	3	0.85	15.5	0.85
Over the treatments	72	0.94	4.8	0.94	72	0.96	0.3	0.96	72	0.78	13.3	0.89	

^1^T1: 0.6ETcNM, T2: 0.6ETcSM, T3: 0.6ETcBM, T4: 0.6ETcJM, T5: 0.8ETcNM, T6: 0.8ETcSM, T7: 0.8ETcBM, T8: 0.8ETcJM, T9: 1.0ETcNM, T10: 1.0ETcSM, T11: 1.0ETcBM, T12: 1.0ETcJM [Drip irrigation treatments to meet 100% (1.0ETc), 80% (0.8ETc) and 60% (0.6ETc) crop evapotranspiration (ETc), respectively, under the standard condition^67^; NM, SM, BM and JM are no-mulch, straw mulch, biodegradable plastic and jute agrotextile mulch, respectively].

n, r^2^, RMSE, and d indicate number of observations, coefficient of determination, root mean square error, and the index of agreement respectively.

### Soil–water-storage

AquaCrop satisfactorily predicted SWS in the 400 mm soil profile with r^2^ of 0.78, RMSE of 13.3 mm, and d of 0.89 (Supplementary Fig. S6 and Table 3). However, the model overestimated SWS marginally in jute agrotextile mulch (BM) treatments (Supplementary Fig. S5).

### Aboveground biomass and yield

**Figure 1:**
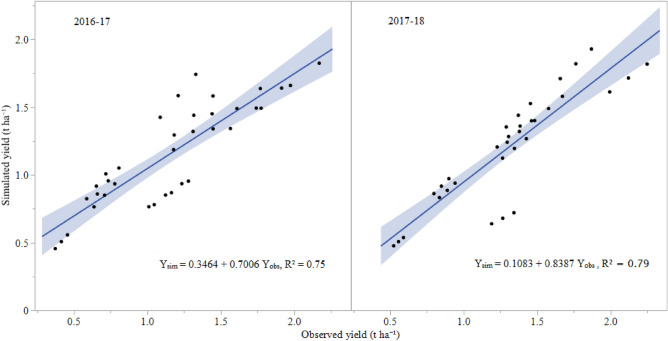
Observed (Y_obs_) and simulated (Y_sim_) fruit yields in strawberry (t ha^−1^) in 2016–17 and 2017–18 seasons.

High r^2^ (0.96) and d (0.96), and low RMSE (0.3 t ha^−1^) values in all the treatments across two seasons of the study demonstrated good agreement with the simulation of the aboveground biomass (Supplementary Fig. S7 and Table 3). The simulated aboveground biomass in this study was lower at maturity under 0.6–0.8ETc irrigation regime irrespective of mulches (Supplementary Fig. S6). However, it predicted the yields satisfactorily in both seasons with r^2^ > 0.92, RMSE ≤ 0.3 t ha^−1^ and d > 0.90 (Fig. 1 and Table 4).

**Table 4:** Validation statistics for the fruit yields of strawberry under different irrigation regimes and mulching during 2016–17 and 2017–18.

Year	N	r^2^	RMSE (t ha^−1^)	d
2016–17	36	0.93	0.3	0.91
2017–18	36	0.92	0.2	0.93
Over the treatments	72	0.93	0.3	0.90

n, r^2^, RMSE, and d indicate number of observations, coefficient of determination, root mean square error, and the index of agreement respectively.

### Irrigation and mulching scenarios analysis

Optimizing the irrigation amount for strawberry requires an understanding of the response of berry productivity (ET, yield, and WP) to irrigation amount. On the other hand, the response also depends on the variability in mulch type and soil texture. Here 108 numerical simulations (9 irrigation amounts × 3 initial soil types × 4 mulch materials) were conducted to select the appropriate irrigation amounts under various mulching and soil types.

### Response of ET to irrigation amount

The ET was directly proportional to irrigation to a maximum level (the maximum ET, ET_m_) for all soil types irrespective of the mulches used (Supplementary Fig. S7). Further increment in irrigation water has not been effective for strawberry, indicating a threshold value for maximum economic return.

### Response of strawberry yield to irrigation amount

The responses of strawberry yield to irrigation amount under various soil and mulch types are reported in Supplementary Fig. S9. The relationship was parabolic with r^2^ values of 0.73, 0.76 and 0.85 for clay, clay loam and sandy clay loam soils, respectively. The yield of berries increased to a maximum at irrigation amount of 165 mm for clay soil, 178 for clay loam soil and 190 mm for sandy clay loam soil. In furtherance of irrigation water application, the fruit yield started declining. The highest yield was simulated in the sandy clay loam with 189 mm of drip irrigation under jute agrotextile mulch (Supplementary Fig. S8).

Strawberry yield differed among soil types (p < 0.001) or the use of mulches (p < 0.05) (Table 5). In general, the yield was highest in sandy clay loam and the lowest in clay soil. The effect of mulches in terms of yield followed the order of JM > BM > SM > NM. The highest yield was predicted with JM in sandy clay loam soil (1.82 t ha^−1^) followed by BM in sandy clay loam soil (Supplementary Fig. S8), while, the lowest yield was predicted in unmulched clay soil.

**Table 5:** Test of significance for crop evapotranspiration (ETc), fruit yield and water productivity (WP) in strawberry under the different scenarios of soil texture and mulch.

Variable	df	Probability		
		ETc	Fruit yield	WP
Soil texture	2	**	***	*
Mulch	3	*	*	***
Soil texture × Mulch	6	*	*	NS

*p < 0.05; **p < 0.01; ***p < 0.001.

### Response of WP to irrigation amount

The WP and irrigation amount had typical parabolic relationships (Supplementary Fig. S9). The WP increased sharply with the increase in yield in response to higher irrigation water use. The yield reached a maximum at the average 200 mm irrigation over soil and mulch types, after which it decreased as more water was applied which lowered the WP. The WP was the highest in sandy clay loam. BM-use checked soil evaporation most effectively in comparison to other mulches and resulted in the highest WP. The interactive effect of soil and mulch type predicted the highest WP in sandy clay loam–JM followed by in sandy clay loam–BM followed by clay loam–JM.

### Appropriate irrigation amount

**Figure 2:**
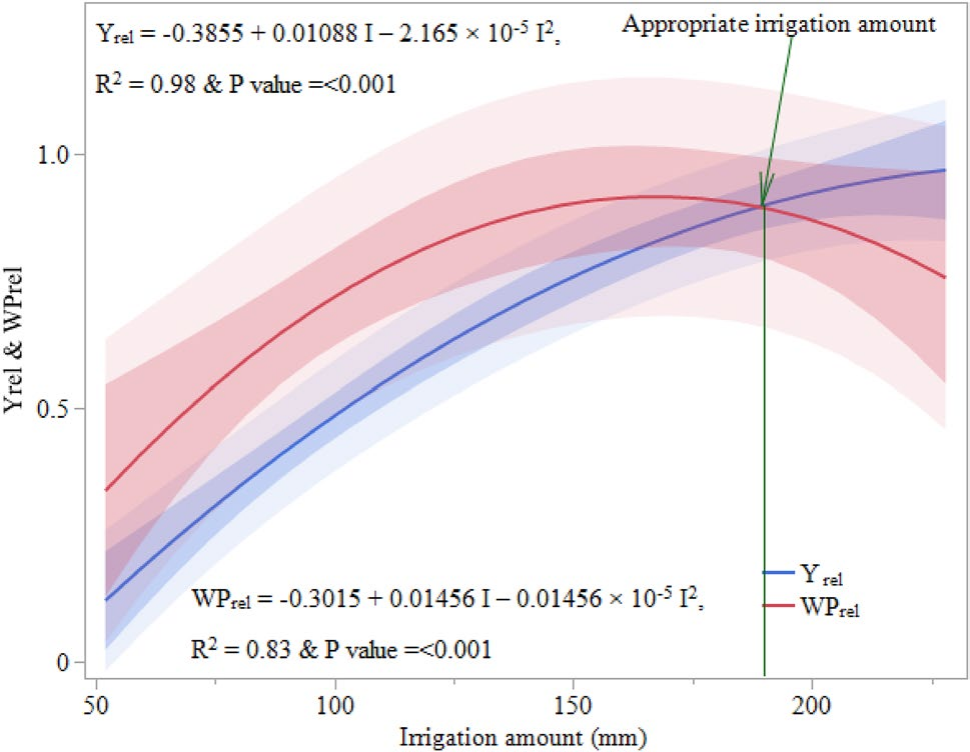
Relative yield (Y_rel_) and water productivity (WP_rel_) of strawberry in response to different irrigation amounts under nonwoven jute agro-textile mulching in sandy clay loam soil.

Optimum irrigation amounts for strawberry ranged between 160 and 200 mm under the test soils and mulch types to minimize loss of water and ensure higher yield and WP. Simulated evapotranspiration (ET_m_) and water productivity (WP) corresponding to the maximum fruit yield (Ym) were generated, and the appropriate irrigation amount was identified under different soil types and also with the use of mulches in strawberry. The appropriate irrigation amounts ranged from 117–145, 137–176 and 153–192 mm for clay, clay loam and sandy clay loam soils, respectively (Table 6). The best yield and WP were achievable with ~ 189 mm irrigation and the use of nonwoven jute agrotextile mulch in sandy clay loam soil (Fig. 2). The differences were most likely caused by different soil textures, and mulch types, that determined the growth and development behaviour of below and above-ground plant parts. The performance of JM was also better compared to other mulches in terms of maximum and appropriate yield, WP and ET. The most optimal irrigation quantities were also found to be greater for sandy clay loam soil than clay loam or clay soil, indicating that strawberry was water sensitive and needed a suitable air–water ratio to ensure both aeration and soil water supply. The ET corresponding to the most appropriate irrigation amount ranged as 126–159, 144–180 and 163–205 mm for clay, clay loam and sandy clay loam soil. Among all soil-mulch combinations, the highest yield was obtained with the jute agrotextile mulch in sandy clay loam (2.78 t ha^−1^) followed by in clay loam soil (2.11 t ha^−1^) with applications of 189 and 176 mm of drip irrigation, respectively.

**Table 6:** Simulated evapotranspiration (ET_m_) and water productivity (WP) corresponding to the maximum fruit yield (Ym) in strawberry, and the same under the best irrigation practice.

Topography	Mulch^1^	Y_m_ (t ha^−1^)	WP_m_ (kg ha^−1^ mm^−1^)	ET_m_ (mm)	Best irrigation practice			
					Amount of irrigation (mm)	Fruit yield (t ha^−1^)	WP (kg ha^−1^ mm^−1^)	ET (mm)
Lowland	NM	1.22	6.9	132	117	1.25	6.3	126
	SM	1.48	7.8	146	122	1.45	7.4	133
	BM	1.65	10.6	152	140	1.73	9.8	146
	JM	2.08	10.5	179	145	1.92	9.5	159
Medium land	NM	1.55	7.5	153	137	1.62	7.0	144
	SM	1.80	9.8	169	143	1.77	8.6	155
	BM	2.26	11.2	187	163	1.83	10.6	173
	JM	2.48	12.6	191	176	2.11	11.3	180
Upland	NM	1.66	9.1	172	153	1.77	7.1	163
	SM	2.36	11.3	189	169	2.03	9.1	177
	BM	2.64	14.1	199	171	2.24	12.6	180
	JM	2.86	13.9	213	192	2.78	13.3	205

^1^NM, SM, BM and JM are no-mulch, straw mulch, biodegradable plastic and jute agrotextile mulch, respectively.

## Discussion

Calibrated parameters indicated good agreements between observed and model-simulated data as reflected from the r^2^ values for canopy cover, soil–water-storage in the root zone, aboveground biomass, and yield. However, lesser average root depth than that recommended by the model for strawberry could be attributed to the application of mulch and drip irrigation, which kept the upper soil layers moist. Soil water content and root length density were observed to be higher in the topsoil layers and the relatively lower in the deeper layer in other studies also^41,42^. The accuracy of the model is also reported in different crops (in soybean^43,44^, potato^45^, tomato^46^, sugar beet^47^, amaranthus^48^ and cabbage^49^) under different hydro-meteorological conditions globally. Model values for canopy cover were 6.5% lower than the field-measured values, indicating a need for further understanding the weather-plant interactions in the model for simulating root growth, moisture utilization and aboveground canopy expressions. However, the canopy senescence was appropriately estimated. However, the model overestimated soil water storage marginally in jute agrotextile mulch. There could be higher soil water uptake by the crop with enhanced root activity and this perforated textile mulch does not cover the soil surface entirely like plastic mulches, and therefore the chance of surface water escaping to the atmosphere. The extent of moisture loss through soil evaporation and crop transpiration depends on the type of mulches^50,51^. Plastic mulch may reduce evaporation loss by almost sealing the soil-atmosphere interface. The aerobic root zone under jute mulch can enhance root growth^5,52,53^ facilitating higher root water uptake, and conserving the residual soil moisture content. Partitioning of evapotranspiration into evaporation and transpiration may be fine-tuned in AquaCrop based on the FAO56 approach^54^. The study demonstrated good agreement with the simulation of the aboveground biomass and is in consistent with previous studies in potato^55^, cabbage^49^, and soybean^44^. However, lower simulated aboveground biomass in this study at maturity under 0.6–0.8ETc irrigation regime irrespective of mulches may be due to a sudden rise in temperature from the second week of February (average temperature increased to 26.7 °C on 27th February from 18.2 °C on 10th February during the representative year 2001) causing thermal heat stress resulting in a higher rate of senescence compared to that predicted by the model. The model computed the aboveground biomass from Water Productivity (WP) and transpiration from an adjusted crop coefficient. Hence the differences could be due to either a lower simulated WP or transpiration underestimating the root water uptake. The model could not pick up the effect of terminal heat stress on the aboveground biomass. This is the limitation of the model for tropical moist sub-humid climate and further work on this aspect is required.

The ET was the highest in sandy clay loam soil and the lowest in clay soil. The influence of soil texture on ET was also explained earlier^56^. The ET was the lowest in BM and the highest in SM in sandy clay loam and clay loam soil. The difference in evaporation and water-retention capacity was observed under different soil types and the use of different mulches^57^.

The relationship between strawberry yield and irrigation amount under various soil and mulch types was parabolic as observed earlier^58,59^. In general, the yield was highest in sandy clay loam and the lowest in clay soil. This may be due to differences in water retention^60^, nutrient availability^61^ and root growth^62^ that influenced above-ground biomass and yield in strawberry in favour of sandy clay loam texture. The impact of mulches on yield exhibited the sequence: JM > BM > SM > NM. The most substantial yield was anticipated using JM in sandy clay loam soil, succeeded by BM in the same soil type. Conversely, the minimal yield was projected for unmulched clay soil. Strawberry is a shallow-rooted crop that requires frequent but minimal amounts of water to produce quality berries. Higher water retention in clay soil can lead to inadequate aeration in the root zone and nutrient leaching, reducing crop performance^63^. Straw mulching moderate soil hydrothermal regime favourably increases water productivity, and keeps berries healthy by reducing contact with the soil to avoid fruit-rot; black polyethene mulch facilitates root growth, and nutrient uptake, provide soil aggregate stability although adversely affecting soil ecophysiology^5^. The use of plastic mulch in the Bengal basin may further warm the soil at the root zone resulting in root and leaf senescence and thereby may reduce crop yield and water use^64^. Organic mulch like JM improved the soil microclimate in favour of microbial growth, rooting behaviour and availability of nutrients^5^. Our study confirms that cultivation of drip-irrigated strawberry is favourable both in sandy clay loam and clay loam soils with the use of organic jute agrotextile mulch for higher yield at appropriate irrigation levels. The combined influence of soil and mulch type indicated the peak water productivity (WP) in sandy clay loam – JM, succeeded by sandy clay loam – BM, and subsequently by clay loam – JM.

The appropriate irrigation amounts worked out under different soil type and mulch type. Y_rel_ and WP_rel_ responded to irrigation amount similarly to yield and WP, and their interactions could likewise be explained by a quadratic function of irrigation amount^65^. The disparities were likely a result of varying soil textures and mulch varieties, influencing the growth and behaviour of both above-ground and below-ground plant components. Additionally, JM outperformed other mulch types in achieving superior outcomes contributing maximum yield, higher water productivity, and controlling evapotranspiration. Furthermore, the ideal irrigation volumes were observed to be higher for sandy clay loam soil in comparison to clay loam or clay soil. This suggests the water sensitivity of strawberries, emphasizing the need for an appropriate air–water balance to ensure effective aeration and adequate soil moisture availability.

## Conclusions

AquaCrop was calibrated using field experimental data (2015–16) and validated with two seasons of data of 2016–17 and 2017–18 to predict canopy cover, soil water storage and above-ground biomass of drip irrigated strawberries in arsenic-affected Bengal basin. Crop and management parameters and their coefficients were adjusted using field data under different irrigation regimes and mulch use. The calibrated model can reliably simulate CC, soil water storage and aboveground biomass in subsequent two seasons in strawberry. Following validation, AquaCrop simulations were run under several treatment conditions (9 irrigation amounts × 3 initial soil types × 4 mulch materials) for drip-irrigated strawberry. The results of these simulations revealed that soil type and mulch had separate and combined effects on ET, yield, and WP. The results on yield, ET and WP revealed that sandy clay loam and clay loam soils are most suitable for strawberry cultivation for upland and medium land, respectively. Simulated irrigation amounts of 176 mm for clay loam and 189 mm for sandy clay loam are the most appropriate. Organic mulch from locally available jute agrotextile may improve 1.4 times higher yield and 1.7 times higher water productivity than that of without mulch in strawberry. The findings of present paper may help all the stakeholders including policymakers to opt for strawberry as alternative more remunerative crop with less water requirement. It can replace groundwater exhaustive winter rice in non-traditional up and medium land of arsenic-affected Bengal basin.

## Methods

### Experimental site

The experiment was conducted in sandy loam soil under a tropical moist sub-humid climate at the Central Research Farm, Bidhan Chandra Krishi Viswavidyalaya, Gayeshpur, India (23°5.3′ N, 83°5.3′ E; 9.75 m above mean sea level). The experimental site is a part of the larger Bengal basin where arsenic contamination in soils and groundwater is predominant. The annual rainfall is 1600 mm of which 85% is received between the 3rd week of June to the end of September. January is the coldest (15.5 to 21.3℃) (Supplementary Table S2), while May is the hottest month (27.6–31.7 °C). Mean relative humidity remains high (82–95%) from June to October and reduces to 70% in January. Wind speed varies from 0.6 to 6.8 km d^−1^. Monthly weather parameters of the growing seasons are given in Supplementary Table S2.

The soil is classified as AericHaplaquept, with the following properties of the surface soil (0–15 cm): pH 6.36 (1:2.5 soil: water), organic carbon 5.2 g kg^−1^ (Walkley–Black), available N (Kjeldahl), P_2_O_5_ (Bray-1)_,_ and K_2_O ((1N NH_4_-acetate) as 89, 13.3, and 65 kg ha^−1^, respectively. Major soil hydrophysical characteristics are listed in Supplementary Table S1.

### Experimental setup

Micro propagated plantlets of a short-day and early strawberry cv Sweet Charlie (FL 80–4925), a cross of FL 80–456 and Pajero was arranged from S.B. Agritech, Satara, Maharashtra, India. It exhibits resistant to anthracnose crown & fruit rot disease. Plantlets were transplanted on October 14, 21, and 16 in the years 2015, 2016, and 2017, respectively, onto trapezoidal raised beds with dimensions of 110 cm at the base, 70 cm at the top, and a height of 30 cm. Spacing was 40 cm between two such trapezoidal beds. There were 40 plants (ten plants per row) in each 3 m × 3 m plot. The experiment was conducted in a randomized complete block design with four replications. There were twelve treatment combinations with three drip irrigation regimes: (i) compensating 100% loss of actual evapotranspiration (1.0ETc), (ii) compensating 80% loss of actual evapotranspiration (0.8ETc), and (iii) compensating 60% loss of actual evapotranspiration (0.6ETc); and four mulch practices: (i) no-mulch (NM), (ii) rice straw mulch at 5 Mg ha^−1^ (SM), (iii) biodegradable mulch of 20 μm thickness (BM), and (iv) nonwoven jute agrotextile mulch with 350 g m^−2^ (gsm) thickness (JM). BM (Ecovio ® M2351; BASF India Ltd., Mumbai, India) is a semi-crystalline, aliphatic and aromatic co-polyester mulch material based on monomers of 1, 4 butanediols, adipic acid, and terephthalic acid in the polymer chain. JM was developed by the ICAR-National Institute of Natural Fibre Engineering and Technology (Erstwhile ICAR-NIRJAFT), Kolkata, West Bengal^30^. The cellulose, hemicellulose, lignin, nitrogenous matter, ash, fat, and wax contents were 60.0, 23.0, 13.0, 1.8, 0.7, and 1.1% in JM and 37.0, 26.0, 10.3, 1.0, 7.2, and 5.4% in SM, respectively^66^. Chopped straw (70 mm length) of rice variety Shatabdi (IET 4786) containing 6.1% Si, 51.2% OC, 0.65% nitrogen, 0.26% phosphorus, and 0.39% potassium was used as straw mulch material.

The date of planting and irrigation scheduling under each irrigation regime and irrigation-mulch combination treatment are presented in Supplementary Fig. S1 and Table 1. Cultivation input details are presented in Supplementary Table S3. JM and BM were placed in each plot with 80 mm diameter slits at 350 mm × 300 mm spacing. Micro-propagated plantlets of strawberry were planted on November 4, November 2, and November 1 in 2015, 2016, and 2017, respectively, on raised beds measuring 1100 mm at the base, 700 mm at the top, and 300 mm in height, with 400 mm spacing between two successive beds. The impermeable film was embedded in the soil at a depth of 600 mm between each plot to prevent lateral seepage.

### Data collection and calculations

#### Meteorological data

Daily meteorological data for rainfall, sunshine hour, maximum and minimum air temperatures, relative humidity, and wind speed were collected from the automatic weather station located approximately 40 m distant from the experimental field. The pan evaporation (Epan, mm) was recorded daily using a standard USWB Class A evaporimeter. The crop reference evapotranspiration (ET0, mm) was computed by multiplying Epan with the pan coefficient (Kp) value. The Kp was taken as 0.85 since the experimental site has > 75% relative humidity, > 2 m s^−1^ wind speed and 1000 m windward side distance from the pan placed in a short green cropped area^67^.

### Measurement of soil–water content

Soil water content (SWC,% v/v) was monitored daily using a PR2/6 profile probe (Delta-T Devices Ltd., Cambridge, UK) from 0–100, 100–200, and 200–300 mm layers before and after each irrigation. Tensiometers were put at 250 and 350 mm soil depths in each plot to estimate drainage from the active root zone (> 90% of the strawberry roots are located within the top 300 mm layer^68^).

### Plant growth parameters

The days after planting (DAP) of first runner development, flowering, fruiting and maturity were recorded. Three plants were chosen at random from each plot to measure the maximum length and width of green leaves every 10–15 days during the growing season.

The individual plants were cut above the ground at 30 days intervals, and the area of the leaves was determined by using an AM 300 leaf area meter (ADC Bio Scientific Ltd., UK) and the leaf area index (LAI) was computed accordingly. The canopy cover (CC) was estimated by using the following equation^69^:

1 $${\text{CC}}=1.005 \times {\left\{1-{\text{exp}}\left(-0.6\mathrm{ LAI }\right)\right\}}^{1.2}$$

Three plants were oven-dried at 70 °C to constant weights^70^ to determine the aboveground biomass. Strawberry fruits were harvested at maturity. There were 13 pickings in the first year and 11 harvests in the second and in the third year at 2 to 7 days intervals. Ten innermost plants from each plot were tagged for recording yield (g plant^−1^) at maturity.

### Model calibration and validation

The AquaCrop model (version 6.1) was selected in this study. Standard values for a few universal parameters in strawberry were considered^71^. Others parameters were required to be adjusted to the local conditions.

Crop parameters for plant development and berry production, as well as soil texture, and hydrological properties were used for model calibration as per the requirement of the model^71^. The slope of aboveground biomass versus normalized transpiration was used to calculate normalized biomass water productivity (i.e., $\sum ({{\text{T}}}_{{\text{r}}}/{{\text{ET}}}_{{\text{o}}}$), T_r_ represents transpiration). The model was calibrated with measured data for all treatments in the 2015–16 season. The mulch coverage percentage was set at 90% (BM and JM), 60% (SM) and 0% (NM). The phenology of the crop was taken as the average of the three growing seasons: 60DAP for flowering, 70DAP for maximum root depth, 79DAP for maximum canopy, 120DAP for senescence, and 140DAP for maturity. The duration of the flowering and yield formation was kept as 60 and 70 days, respectively. Measured SWC at sowing was taken as the initial input to the model. The calibrations were run in day mode, beginning by fitting the parameters from 1.0ETcSM plots (drip irrigation at 100% evaporative demand and use of straw mulch). The crop parameters at 1.0ETcSM treatment were repeatedly altered until the simulated and observed results (SWS, CC, above-ground biomass, and yield) matched acceptably well for other treatments in 2015–16. The main crop parameters in AquaCrop for drip irrigated and mulched strawberry are presented in Supplementary Table S4.

After the calibration, the model was validated using the measured data sets from the 2016–17, and 2017–18 seasons. The model's outputs were compared to the observed data using the coefficient of determination (r^2^), root mean square error (RMSE), and the index of agreement (d), which were calculated as:

2 $${r}^{2}={\left(\frac{\sum_{i=1}^{n}({{\text{O}}}_{i}-\overline{{\text{O}}})({{\text{S}}}_{i}-\overline{\mathrm{S })}}{\sqrt{\sum_{i=1}^{n}({{\text{O}}}_{i}{-\overline{{\text{O}}})}^{2}\sum_{i=1}^{n}{({{\text{S}}}_{i}-\overline{\mathrm{S })}}^{2}}}\right)}^{2}$$

3 $${\text{RMSE}}=\sqrt{\frac{1}{n}\sum_{i=1}^{n}{\left({{\text{S}}}_{{\text{i}}}-{{\text{O}}}_{{\text{i}}}\right)}^{2}}$$

4 $${\text{d}}=1- \frac{\sum_{i=1}^{n}{\left({{\text{S}}}_{{\text{i}}}-{{\text{O}}}_{{\text{i}}}\right)}^{2}}{\sum_{i=1}^{n}{\left(\left|{{\text{S}}}_{{\text{i}}}-\overline{{\text{O}}}|\right|+|{{\text{O}}}_{i}-\overline{{\text{O}}}|\right)}^{2}}$$

where Oi and Si are the observed and simulated values, respectively; O and S are the average observed and simulated values, respectively; and n is the number of observations. The r^2^ represents the proportion of the variance in measured data explained by the model. The average magnitude of the discrepancy between simulations and observations is measured by the RMSE, where a maximum 15% error is acceptable for agronomic studies^72,73^. The index of agreement (d) is a measure of the over- and under-estimations of the model, which can’t be judged by r_2_^74^. It has been reported that the results from the simulation may be acceptable at r^2^ > 0.5 or d > 0.65^75^.

### Simulation scenario

After the validation, the model was used to simulate ETc, yield, and WP of strawberry under various irrigation amounts, and to identify the best irrigation and mulch treatment in clay soil (1.2–4.4% sand, 30.5–32.7% silt and 65.1–66.1% clay) of lowland (0–30 m amsl; traditional winter rice area), clay loam (11.0–12.8% sand,35.3–43.3% silt and 43.9–53.7% clay) of medium land (31–60 m amsl; non-traditional upland arsenic contaminated winter rice area) and sandy clay loam (42.4–42.7% sand, 20.7–26.8% silt and 30.5–36.9% clay) of upland (61–100 m amsl; mon-traditional medium land arsenic contaminated area areas of Bengal basin^2^. Unbridled groundwater extraction is prevalent in these two non-traditional rice areas^1,5,76^. The simulations were run using a groundwater depth of 2.0 m. The climate in the year 2000-01 was selected as the representative of the long-term average based on the average Mahalanobis distance obtained from variance–covariance matrices (Supplementary Fig. S2). This data was used to simulate the scenarios in AquaCrop.

The sowing date of strawberry was taken as 7th October in the simulations of the scenarios. The long-term average ETc was 140 mm (Kc was 0.40, 0.85 and 0.75 during 1–30, 31–80 and 81–140DAP, respectively;^67^ Allen et al., 1998). The simulations were performed for irrigation amounts ranging between 60 and 220 mm with 20-mm increments {nine irrigation amounts 60 mm (to meet 43% of crop evaporative demand or 0.43ETc), 80 (0.57ETc), 100 (0.71ETc), 120 (0.86ETc), 140 (1.0ETc), 160 (1.14ETc), 180 (1.29ETc), 200 (1.43ETc) and 220 mm (1.57ETc)}, covering deficit (< 140 mm), full (140 mm), and over-irrigation (> 140 mm) through a drip system to determine the response of water productivity to irrigation amount.

A total of 14 irrigation events with the same irrigation frequency and irrigation amount after adjusting the effective rainfall and ETc were set for the simulation of each scenario. The first and the last irrigation was given on 17 and 136DAP, respectively, along with an additional 25 mm water for land preparation and seedling establishment up to 15DAP. A total of 108 numerical simulations (9 irrigation amounts × 3 initial soil types × 4 mulch materials) were carried out.

The analysis of variance was used to investigate the differences in simulated ETc, yield, and WP (Eq. 5) in strawberry under different scenarios (soil types, irrigation and mulch materials). Relative yields (Y_rel_; Eq. 6) and relative WPs (WP_rel_; Eq. 7)^65^ were also used to identify the best irrigation requirements to obtain high yields and WP under the scenarios.

5 $${\text{WP}}= \frac{100\mathrm{ Y}}{{\text{ET}}}$$

6 $${{\text{Y}}}_{{\text{rel}}}= \frac{{\text{Y}}}{{{\text{Y}}}_{{\text{m}}}}$$

7 $${{\text{WP}}}_{{\text{rel}}}= \frac{{\text{WP}}}{{{\text{WP}}}_{{\text{m}}}}$$

where Y (t ha^−1^) is the simulated yield, Y_m_ (t ha^−1^) is the simulated maximum yield, WP (kg ha^−1^ mm^−1^) is the simulated maximum WP among the scenarios, and ET (mm) is simulated evapotranspiration during the entire crop growth period.

### Ethics approval and consent to participate

The collection and use of plant materials in this study is carried out in accordance with existing National/International/Legislative/Institutional guidelines and regulations and the plant collection and use was in accordance with all the relevant guidelines.

### Supplementary Information

Supplementary Information.

## Data Availability

All data generated or analysed during this study are included in this published article [and its supplementary information files].
