# Supplementary material for: Modelling the impact of different irrigation regimes and mulching on strawberry crop growth and water use in the arsenic-contaminated Bengal basin
Source: Sci Rep. 2024 Apr 26;14:9586. doi: 10.1038/s41598-024-56664-4 (PMC11053059; doi:10.1038/s41598-024-56664-4)
Supplement: Supplementary file 1 — Supplementary Information. [file 41598_2024_56664_MOESM1_ESM.docx]

**Supplementary File**

**Supplementary Table S1** Hydro physical characteristics of the soil

| Soil layers (mm) | Particle size (%) (Hydrometer method) | | | Bulk density  (Mg m^-3^) | Saturated hydraulic conductivity  (mm h^-1^) | Soil water content (mm) | |
| --- | --- | --- | --- | --- | --- | --- | --- |
|  |  | | |  |  | Field capacity | Wilting point |
|  | Sand | Silt | Clay |  |  |  |  |
| 0-100 | 58.7 | 23.4 | 17.9 | 1.4 | 4.3 | 36.9 | 10.3 |
| 100-200 | 46.9 | 33.0 | 20.1 | 1.5 | 1.9 | 37.1 | 14.2 |
| 200-300 | 48.4 | 29.6 | 22.0 | 1.4 | 1.3 | 36.6 | 12.2 |
| 300-400 | 56.4 | 24.1 | 19.5 | 1.7 | 2.8 | 38.5 | 10.3 |

**Supplementary Table S2** Monthly weather parameters during the crop seasons at the experimental site

|  | Crop season | | | | | | | | | | | | | | |
| --- | --- | --- | --- | --- | --- | --- | --- | --- | --- | --- | --- | --- | --- | --- | --- |
|  | 2015-16 | | | | | 2016-17 | | | | | 2017-18 | | | | |
|  | Nov | Dec | Jan | Feb | Mar | Nov | Dec | Jan | Feb | Mar | Nov | Dec | Jan | Feb | Mar |
| Average maximum temperature (°C) | 31.3 | 26.2 | 25.8 | 30.7 | 34.4 | 29.7 | 26.2 | 26.0 | 30.3 | 33.3 | 29.3 | 25.9 | 24.3 | 30.8 | 35.1 |
| Average minimum temperature (°C) | 18.8 | 14.8 | 11.9 | 18.0 | 21.9 | 17.7 | 12.8 | 10.8 | 15.2 | 20.7 | 17.8 | 14.3 | 8.7 | 15.9 | 21.1 |
| Average maximum relative humidity (%) | 93.1 | 93.0 | 92.7 | 92.8 | 91.6 | 93.4 | 93.9 | 91.0 | 89.9 | 91.7 | 93.1 | 93.6 | 90.8 | 90.0 | 89.4 |
| Average minimum relative humidity (%) | 53.3 | 56.3 | 53.3 | 53.9 | 47.1 | 58.7 | 58.9 | 49.1 | 45.4 | 48.5 | 57.3 | 61.8 | 47.3 | 44.4 | 40.5 |
| Average daily sunshine hour | 6.9 | 3.3 | 4.6 | 5.2 | 7.4 | 6.6 | 5.3 | 6.8 | 6.6 | 7.3 | 7.6 | 6.0 | 6.7 | 6.2 | 7.0 |
| Rainfall (mm) | 0.0 | 6.6 | 3.0 | 31.9 | 35.8 | 16.6 | 0.0 | 0.0 | 0.0 | 8.9 | 37.0 | 15.4 | 0.0 | 0.0 | 1.5 |
| Open pan evaporation (mm) | 49.6 | 32.1 | 30.6 | 53.9 | 92.2 | 40.5 | 31.0 | 36.8 | 58.1 | 99.1 | 47.4 | 30.2 | 36.0 | 58.6 | 105.7 |

**Supplementary Table S3** List of input for strawberry cultivation

| Input | Quantity |
| --- | --- |
|  |  |
| Mustard cake (kg ha^-1^) | 375 |
| Farmyard manure (t ha^-1^) | 25 |
| Fertiliser N (kg ha^-1^) | 80 |
| Fertiliser P (kg ha^-1^) | 40 |
| Fertiliser K (kg ha^-1^) | 40 |
| NPK 15:15:15 (kg ha^-1^) | 30 |
| Zinc (kg ha^-1^) | 5.25 |
| Boron (kg ha^-1^) | 1 |
| Strawberry plantlets (kg ha^-1^) (dry mass) | 80 |
| Triacontanol EC 0.05% (l ha^-1^) | 1 |
| Azadirachtin EC 0.03% (l ha^-1^) | 20 |
| Seaweed extract 15% v/v (l ha^-1^) | 4 |
| Chlorpyriphos 20% EC (l ha^-1^) | 4 |
| Carbendazim 50% WP (kg ha^-1^) | 4 |
| Fenpyroximate 5% EC (l ha^-1^) | 4 |
| Metiram 55% + Pyraclostrobin 5% WG (kg ha^-1^) | 2.5 |
| Carbosulfan 25% EC (l ha-1) | 2 |
| Thiodicarb 75% WP (kg ha-1) | 1.5 |
| Diesel (l ha^-1^) | 7.5 |
| Machinery (h ha^-1^) | 15 |
| Labour before harvest (8-h days ha^-1^) | 131.5 |
| Labour for surface irrigation (8-h days ha^-1^) | 40 |
| Labour for drip irrigation (8-h days ha^-1^) | 125 |
| Straw mulch (t ha^-1^) | 5 |
| Nonwoven jute agrotextile 350 gsm mulch (t ha^-1^) | 3.5 |
| Biodegradable plastic film 20μm (kg ha^-1^) | 200 |
| Black polyethylene 50μm (kg ha^-1^) | 500 |
| Labour for harvest and processing per ton economic product (8-h days t^-1^) | 22.95 |

**Supplementary Table S4** Key crop parameters of strawberry in AquaCrop model under straw-mulched drip irrigation at 1.0ETc

| Parameter | Description | Value | Unit | Remarks^3^ |
| --- | --- | --- | --- | --- |
| *Canopy development and production* | | | | |
| CC_o_ | Initial canopy cover | 0.80 | % | Measured |
| CCx | Maximum canopy cover | 95.00 | % | Measured |
| CGC | Canopy-growth coefficient | 10.4 | % d^-1^ | Calibrated |
| CDC | Canopy-decline coefficient | 8.00 | % d^-1^ | Calibrated |
| Z_n_ | Minimum effective root growth | 0.30 | m | Measured |
| Z_m_ | Maximum effective root growth | 0.40 | m | Measured |
| f_shape,z_ | Shape factor for root-zone expansion | 1.50 |  | Recommended |
| WP | Water productivity normalised for ET_0_ and CO_2_ | 17.00 | g m^-2^ | Measured |
| f_yield_ | Water productivity normalised for ET_0_ and CO_2_ during yield formation | 40.00 | % | Calibrated |
| f_CO2_ | Crop performance under elevated atmospheric CO_2_ concentration | 50.00 | % | Calibrated |
| HI_o_ | Reference harvest index | 35.00 | % | Calibrated |
| S_m_ | Maximum root-water extraction over an effective root zone^1^ | 12.30 | mm d^-1^ | Measured |
| KcTr,x | Crop coefficient at CC =100% prior to senescence | 1.10 |  | Recommended |
| *Water-stress response* | | | | |
| P_exp.upper_ | Fraction of TAW^2^ at which canopy expansion is limited | 0.20 |  | Calibrated |
| P_exp.lower_ | Fraction of TAW at which canopy expansion stops | 0.55 |  | Calibrated |
| F_exp,w_ | Shape factor for water-stress coefficient of canopy expansion | 3.00 |  | Recommended |
| p_sto_ | Fraction of TAW at the beginning of stomatal closure | 0.50 |  | Calibrated |
| F_shape,sto_ | Shape factor for water-stress coefficient of canopy senescence | 3.00 |  | Recommended |
| p_sen_ | Fraction of TAW at the beginning of early canopy senescence | 0.65 |  | Calibrated |
| F_shape,sen_ | Shape factor for water-stress coefficient of canopy senescence | 3.00 |  | Recommended |
| p_pol_ | Fraction of TAW at the beginning of pollination failure | 0.90 |  | Recommended |

^a^From ^1^; ^b^ Total available soil water (between field capacity and wilting point) in the root zone; ^3^Calibrated values using 2015-16 field-measured data (‘Calibrated’) and values recommended in the AquaCrop manual (‘Recommended’).

| Cumulative irrigation (mm) |  |
| --- | --- |
|  |  |
|  |  |
|  | Days after planting |
| **Supplementary Fig. S1** Irrigation schedule based on crop ET (ETc) demand [Drip irrigation treatments to meet 100% (1.0ETc), 80% (0.8ETc) and 60% (0.6ETc) crop evapotranspiration (ETc), respectively, under the standard condition ^2^] | |

| 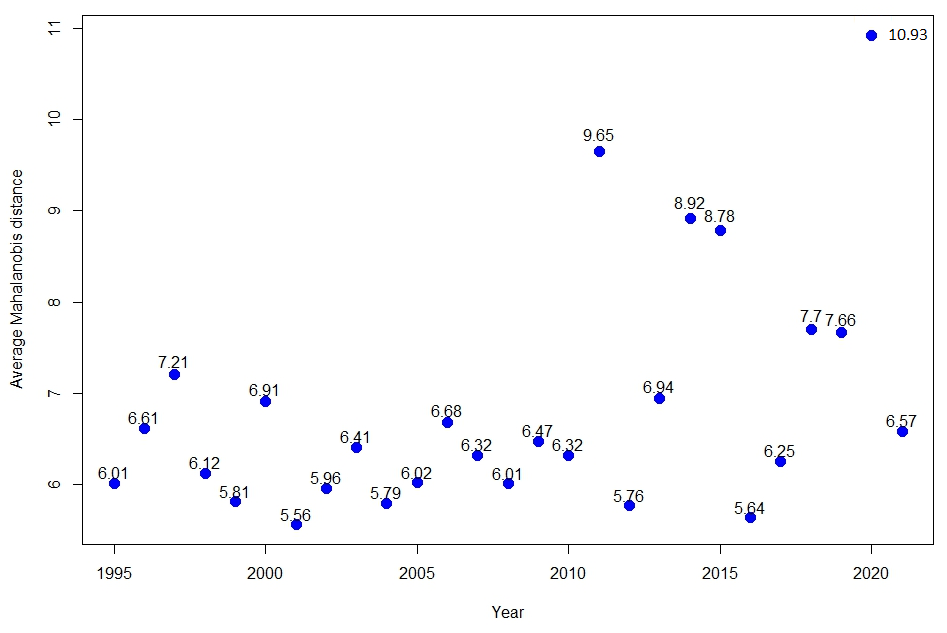 |
| --- |
| **Supplementary Fig. S2** Temporal variations of yearly average Mahanalobis Distance during 1995-2021. |

| 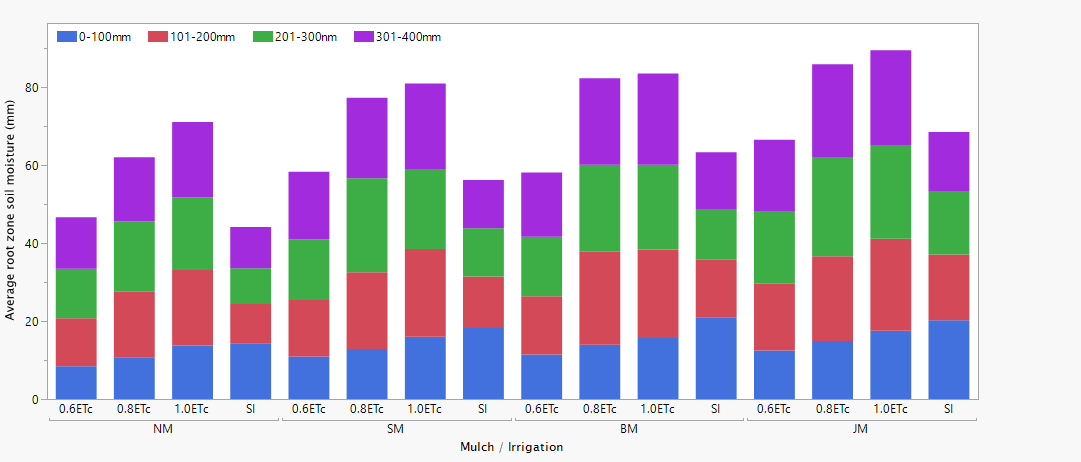 |
| --- |
| **Supplementary Fig. S3** Average soil moisture storage in the active root zone of strawberry during 2015-16.  NM, SM, BM and JM are no-mulch, straw mulch, biodegradable plastic and jute agrotextile mulch, respectively.  SI is surface irrigation at IW/CPE = 1 and drip irrigation treatments to meet 100, 80 and 60% crop evapotranspiration (ETc) are 1.0ETc, 0.8ETc and 0.6ETc, respectively |

| 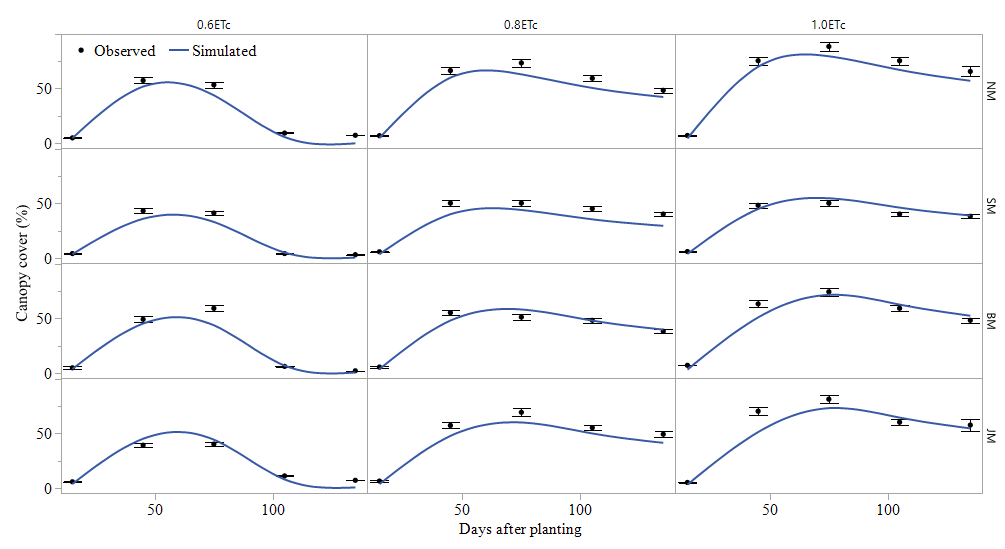 |
| --- |
| **Supplementary Fig. S4a** Observed and simulated canopy cover percentage of strawberry under different irrigation regimes and mulching during 2016-17. Error bars represent standard deviations  NM, SM, BM and JM are no-mulch, straw mulch, biodegradable plastic and jute agrotextile mulch, respectively.  Drip irrigation treatments to meet 100, 80 and 60% crop evapotranspiration (ETc) are 1.0ETc, 0.8ETc and 0.6ETc, respectively |

| 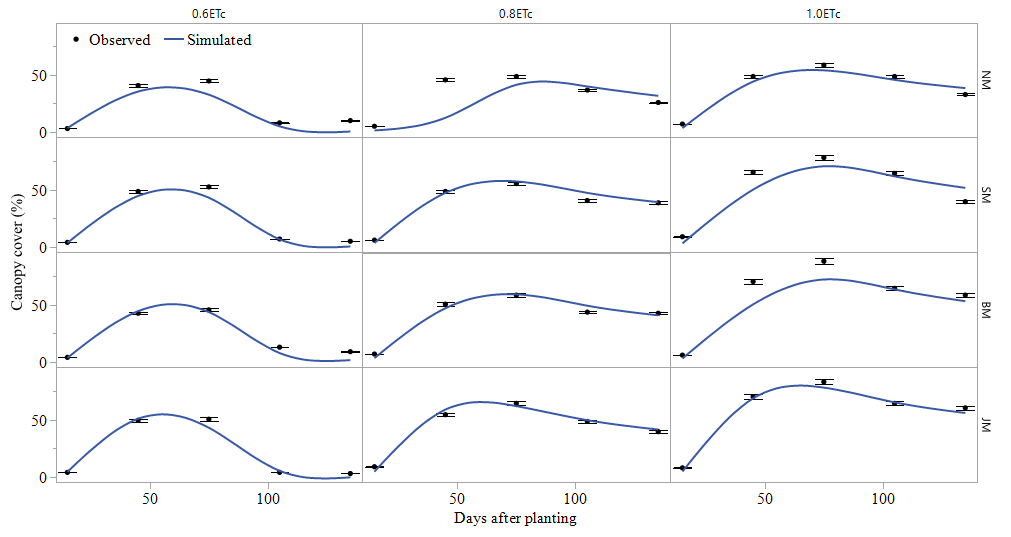 |
| --- |
| **Supplementary Fig. S4b.** Observed and simulated canopy cover percent of strawberry under different irrigation regimes and mulching during 2017-18. Error bars represent standard deviations  NM, SM, BM and JM are no-mulch, straw mulch, biodegradable plastic and jute agrotextile mulch, respectively.  Drip irrigation treatments to meet 100, 80 and 60% crop evapotranspiration (ETc) are 1.0ETc, 0.8ETc and 0.6ETc, respectively |

| 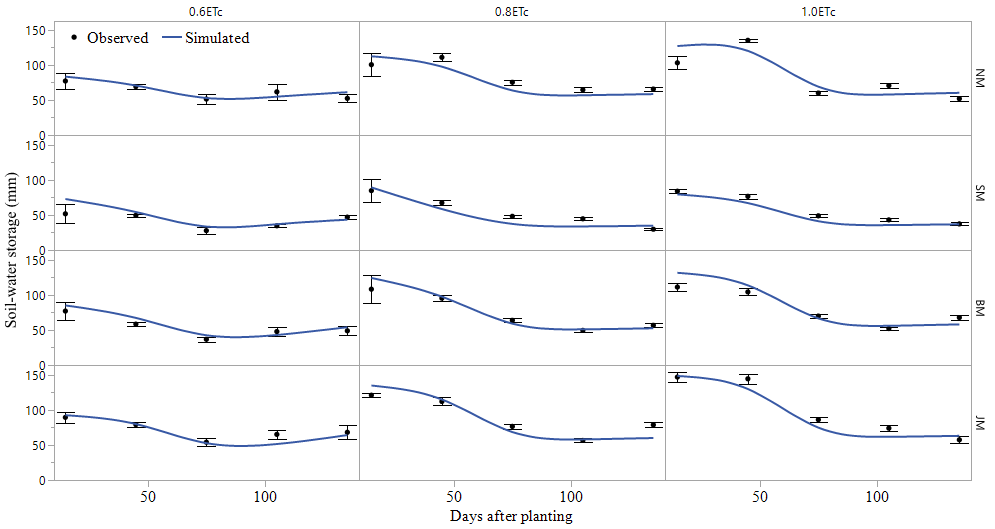 |
| --- |
| **Supplementary Fig. S5a.** Observed and simulated soil-water storage (mm) at active root zone (0.4m) of strawberry under different irrigation regimes and mulching during 2016-17. Error bars represent standard deviations  NM, SM, BM and JM are no-mulch, straw mulch, biodegradable plastic and jute agrotextile mulch, respectively.  Drip irrigation treatments to meet 100, 80 and 60% crop evapotranspiration (ETc) are 1.0ETc, 0.8ETc and 0.6ETc, respectively |

| 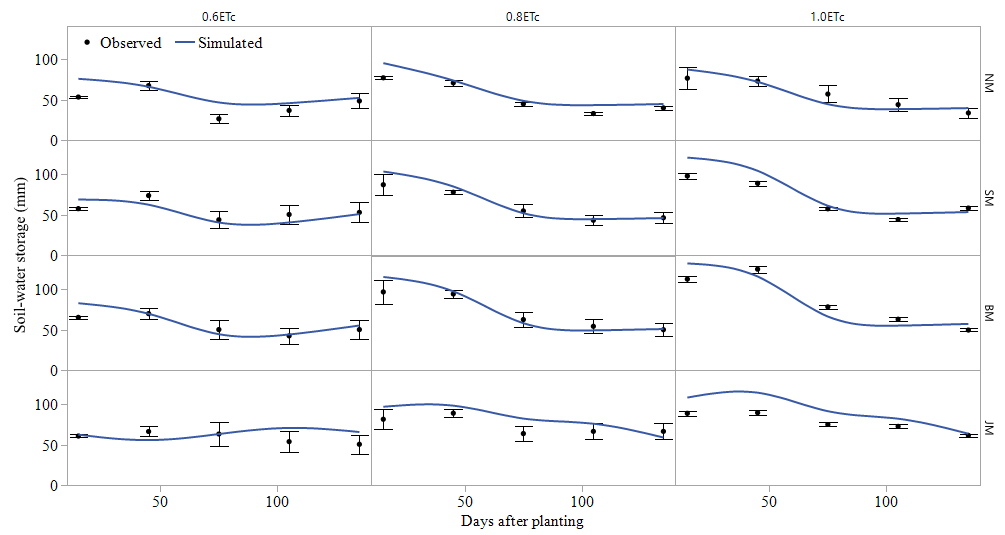 |
| --- |
| **Supplementary Fig. S5b.** Observed and simulated soil-water storage (mm) at active root zone (0.4m) of strawberry under different irrigation regimes and mulching during 2017-18. Error bars represent standard deviations.  NM, SM, BM and JM are no-mulch, straw mulch, biodegradable plastic and jute agrotextile mulch, respectively.  Drip irrigation treatments to meet 100, 80 and 60% crop evapotranspiration (ETc) are 1.0ETc, 0.8ETc and 0.6ETc, respectively |

| 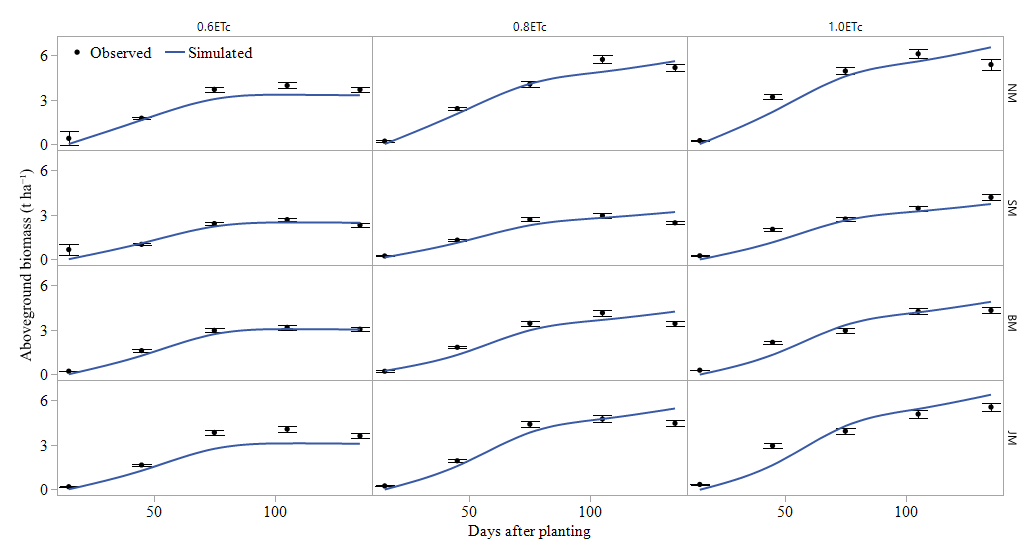 |
| --- |
| **Supplementary Fig. S6a.** Observed and simulated aboveground biomass (t ha^-1^) of strawberry under different irrigation regimes and mulching during 2016-17. Error bars represent standard deviations  NM, SM, BM and JM are no-mulch, straw mulch, biodegradable plastic and jute agrotextile mulch, respectively.  Drip irrigation treatments to meet 100, 80 and 60% crop evapotranspiration (ETc) are 1.0ETc, 0.8ETc and 0.6ETc, respectively |

| 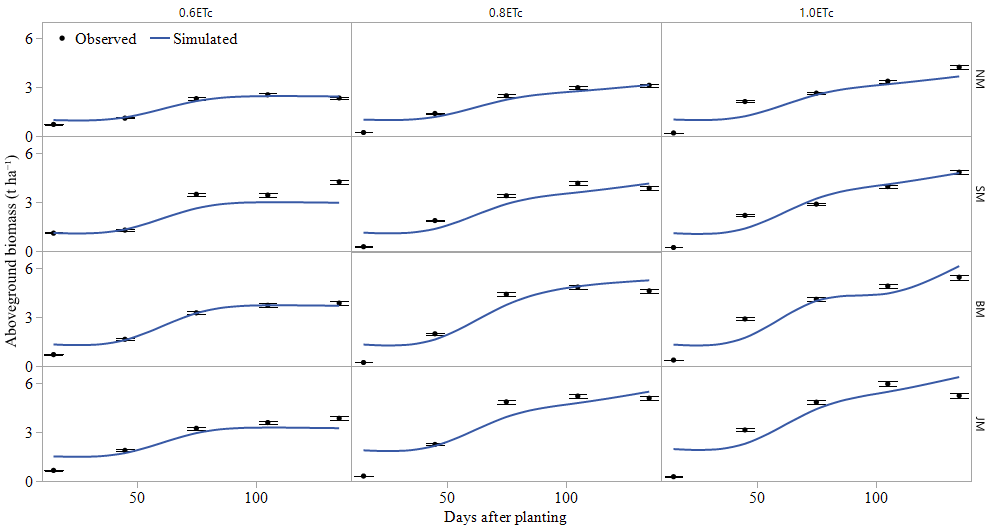 |
| --- |
| **Supplementary Fig. S6b.** Observed and simulated aboveground biomass (t ha^-1^) of strawberry under different irrigation regimes and mulching during 2017-18. Error bars represent standard deviations  NM, SM, BM and JM are no-mulch, straw mulch, biodegradable plastic and jute agrotextile mulch, respectively.  Drip irrigation treatments to meet 100, 80 and 60% crop evapotranspiration (ETc) are 1.0ETc, 0.8ETc and 0.6ETc, respectively |

| 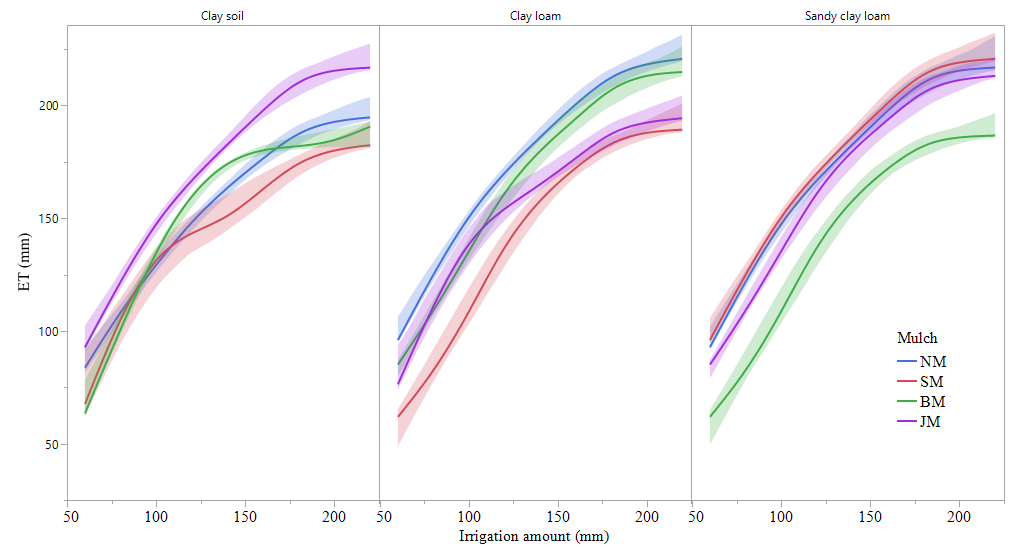 |
| --- |
| **Supplementary Fig. S7.** The relationship between evapotranspiration (ET) and irrigation amount under scenarios of different soil texture and mulch materials; NM, SM, BM and JM are no-mulch, straw mulch, biodegradable plastic and jute agrotextile mulch, respectively. |

| 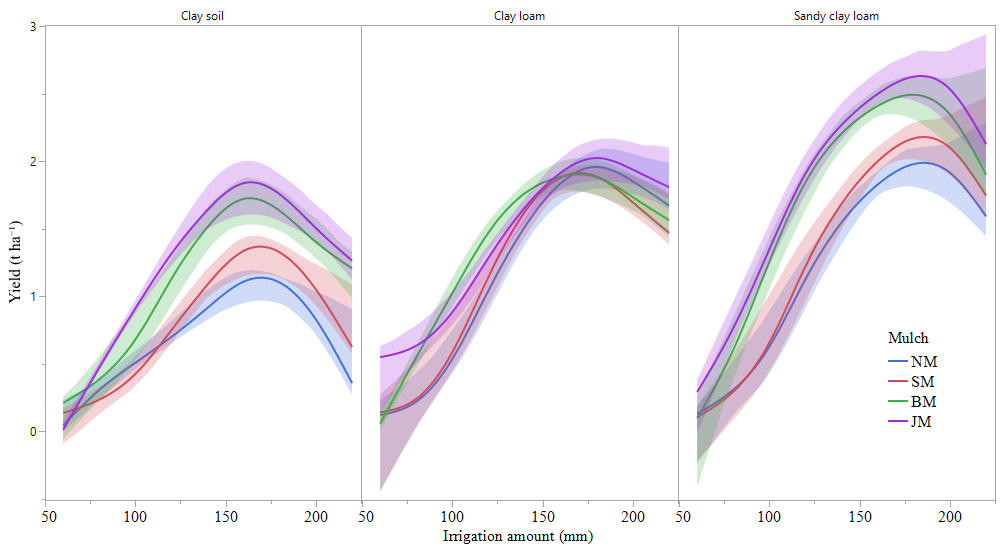 |
| --- |
| **Supplementary Fig. S8.** The relationship between strawberry yield and irrigation amount under scenarios of different soil textures and mulch materials; NM, SM, BM and JM are no-mulch, straw mulch, biodegradable plastic and jute agrotextile mulch, respectively. |

| 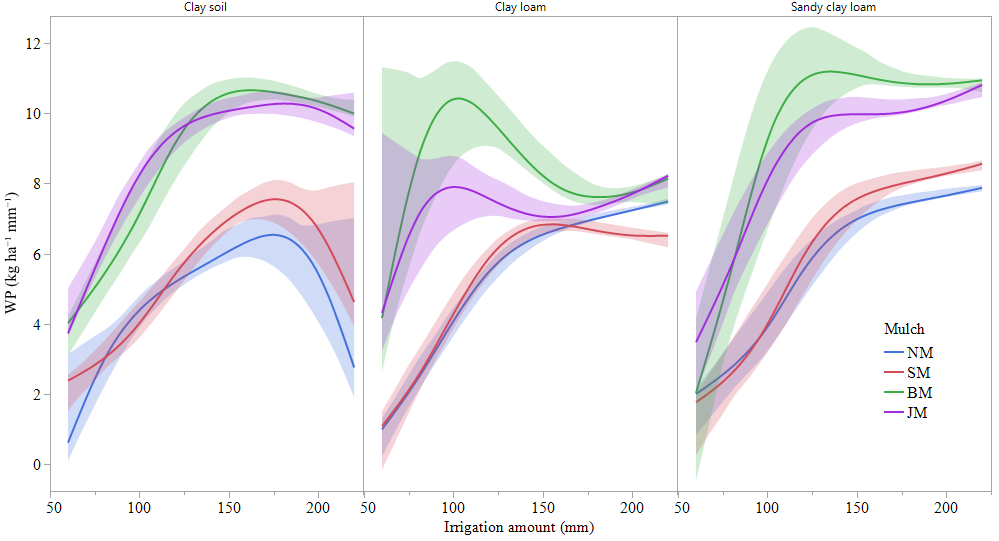 |
| --- |
| **Supplementary Fig. S9.** The relationship between water productivity of strawberry and irrigation amount under scenarios of different soil textures and mulch materials; NM, SM, BM and JM are no-mulch, straw mulch, biodegradable plastic and jute agrotextile mulch, respectively. |

**Reference:**

1. Biswas, B. *et al.* Replacing winter rice in non-traditional areas by strawberry reduces arsenic bioaccumulation , and improves water productivity and profitability. *Science of the Total Environment* **788**, 147810 (2021).

2. Allen, R. G., Pereira, L. S., Raes, D. & Smith, M. *Crop Evapotranspiration. Guidelines for Computing Crop Water Requirements. FAO Irrigation and Drainage Paper, No. 56*. (Food and Agricultural Organization, Rome., 1998).
